# FIB-SEM tomography of human skin telocytes and their extracellular vesicles

**DOI:** 10.1111/jcmm.12578

**Published:** 2015-03-30

**Authors:** Dragos Cretoiu, Mihaela Gherghiceanu, Eric Hummel, Hans Zimmermann, Olga Simionescu, Laurentiu M Popescu

**Affiliations:** a Department of Cellular and Molecular Medicine, Carol Davila University of Medicine and PharmacyBucharest, Romania; b Victor Babeş National Institute of PathologyBucharest, Romania; c Carl Zeiss Microscopy, GmbHMunich, Germany; d Department of Dermatology, Colentina University Hospital, Carol Davila University of Medicine and PharmacyBucharest, Romania

**Keywords:** dermis, FIB-SEM tomography, telocytes, telopodes, extracellular vesicles, scleroderma, multiple sclerosis

## Abstract

We have shown in 2012 the existence of telocytes (TCs) in human dermis. TCs were described by transmission electron microscopy (TEM) as interstitial cells located in non-epithelial spaces (stroma) of many organs (see www.telocytes.com). TCs have very long prolongations (tens to hundreds micrometers) named Telopodes (Tps). These Tps have a special conformation with dilated portions named podoms (containing mitochondria, endoplasmic reticulum and caveolae) and very thin segments (below resolving power of light microscopy), called podomers. To show the real 3D architecture of TC network, we used the most advanced available electron microscope technology: focused ion beam scanning electron microscopy (FIB-SEM) tomography. Generally, 3D reconstruction of dermal TCs by FIB-SEM tomography revealed the existence of Tps with various conformations: (*i*) long, flattened irregular veils (ribbon-like segments) with knobs, corresponding to podoms, and (*ii*) tubular structures (podomers) with uneven calibre because of irregular dilations (knobs) – the podoms. FIB-SEM tomography also showed numerous extracellular vesicles (diameter 438.6 ± 149.1 nm, *n* = 30) released by a human dermal TC. Our data might be useful for understanding the role(s) of TCs in intercellular signalling and communication, as well as for comprehension of pathologies like scleroderma, multiple sclerosis, psoriasis, *etc*.

## Introduction

Telocytes (TCs) were described 5 years ago 1 as a new cell type in the interstitial space of many organs 2–23 as well as in human skin 24 (see www.telocytes.com). Their presence in human dermis was confirmed 18,25–27 and their importance for pathology was revealed 28–35. The main differential diagnosis of TCs in human papillary dermis should be made with fibroblasts. Indeed, numerous data suggested there are a lot of differences between TCs and fibroblasts: (*i*) the aspect of TCs and fibroblasts is not the same in tissue culture 36,37; (*ii*) transmission electron microscopy (TEM) shows completely different ultrastructure, *e.g*. 5,18,20,38; (*iii*) microRNA imprint is dissimilar, *e.g*. 39; (*iv*) gene profile are not the same 40–42 and (*v*) proteomics showed striking differences 43.

Focused ion beam scanning electron microscopy (FIB-SEM) is now the election technique for three-dimensional (3D) visualization of biological structures (cells) at nanoscale resolution 44–53. FIB-SEM tomography allows 3D imaging at the subcellular level and is considered a breakthrough for ultrastructural volume reconstruction.

Here, we present FIB-SEM tomography of human papillary dermis TCs showing their complex 3D architecture, as well as the budding and shedding of extracellular vesicles. FIB-SEM tomography does not contradict TEM, but provides additional important details.

## Material and methods

### Sample preparation

Biopsies of human skin were obtained from three patients (informed written consent). Normal skin samples were obtained from a re-excision procedure after removing a local melanoma. The second excisions were performed according to the Breslow index (tumoural depth), 14 days after primary excision. The samples of normal skin were taken at 1-cm distance from primary suture 24. Experiments were performed according to the Helsinki guidelines, in full compliance with the Bioethics Committee of the ‘Victor Babeş’ National Institute of Pathology, Bucharest regulations. The small samples of skin were processed as described previously 12. Briefly, the 1-mm-cube fragments were fixed by immersion in 4% glutaraldehyde, and post-fixed in 1% OsO_4_ with 1.5% K_4_Fe(CN)_6_ (potassium ferrocyanide – reduced osmium) to increase the membranes contrast. Subsequently, the samples were dehydrated through increasing graded ethanol series and embedded in epoxy resin (Agar 100 from Agar Scientific, Essex, UK) at 60°C for 48 hrs.

### FIB/SEM image stack acquisition

Focused ion beam milling and SEM imaging were carried out with a ZEISS Auriga Crossbeam system (from Carl Zeiss Microscopy, München, Germany). FIB milling was performed with 600 pA to 20 nA for the given samples. SEM-Imaging current was 220 pA. To achieve the best signal contrast, the mixed Inlens and energy-selective backscattered detector signals were used. FIB milling steps was 10 nm/slice and each 5th slice was imaged. Accordingly, each image represents 50 nm of the stack, at 9k× magnification. Image pixel size was 10.27 nm.

### Image processing and analysis

Image stack was analyzed and processed using Adobe Photoshop CS5 Extended (Adobe Systems Incorporated, San Jose, CA, USA) for noise detection and removal and luminance level adjustment. Output images were then loaded into Amira 5.0.1 (Visage Imaging, Berlin, Germany) software package. Structures of interest were manually segmented and reconstructed. Stacks of images were also loaded in VirtualDub v1.10.4 (Lee A.) software as sequence of numbered JPEG files and converted to video file. Adobe Photoshop CS5 Extended was also used for vesicles 2D morphometry. Measurements were then statistically analyzed using Microsoft Excel 2013 Analysis ToolPak module.

## Results and discussion

**Fig 1:**
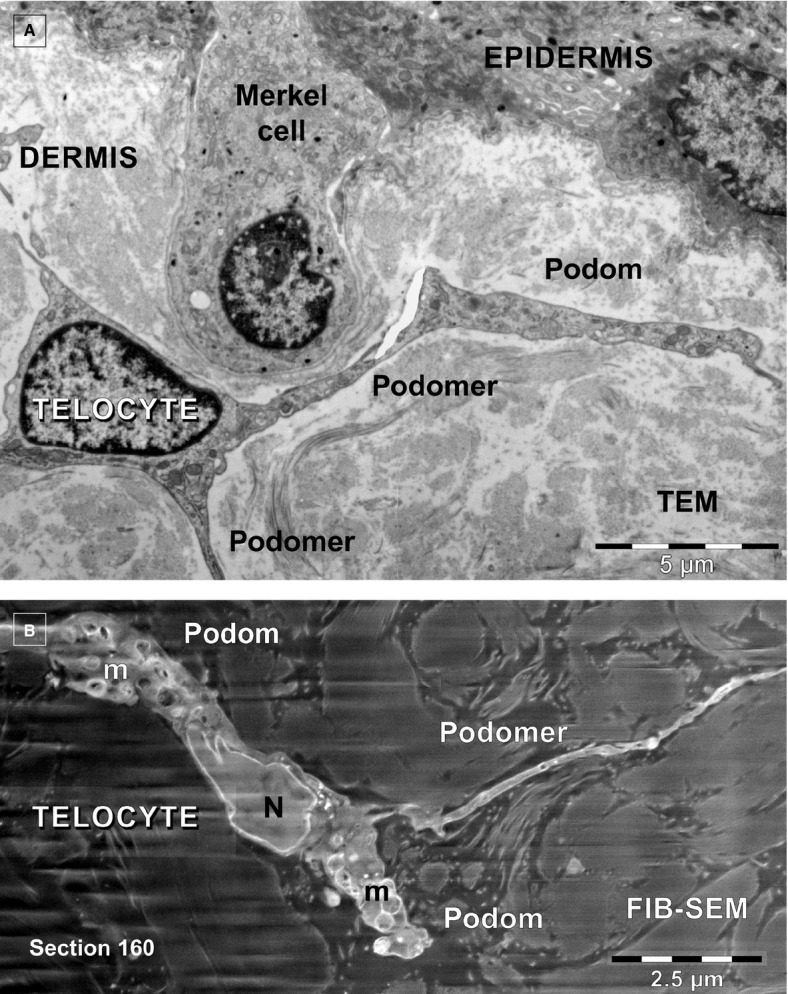
(A and B) Telocytes in human papillary dermis. (A) Transmission electron microscopy shows a telocyte with 3 telopodes edging a Merkel cell. (B) FIB-SEM backscattered electron imaging mode shows a telocyte with two telopodes in dermis.

**Fig 2:**
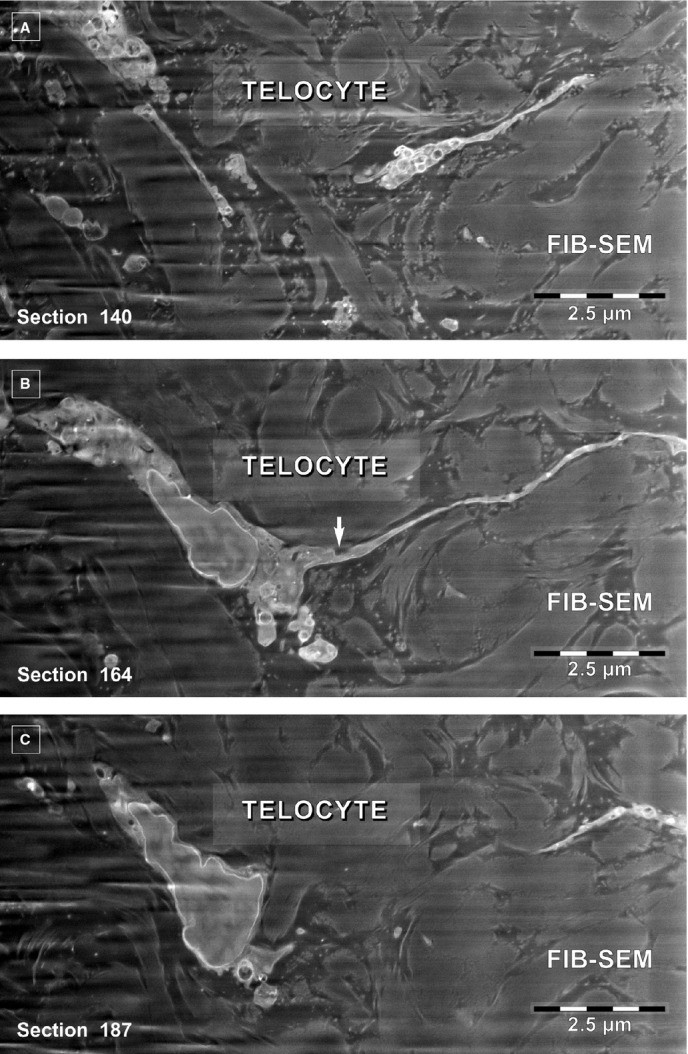
(A–C) FIB-SEM backscattered electron images. Three non-consecutive serial images obtained at ∼1.2 μm z-interval show the narrow emergence (arrow) of a telopode from the cellular body of a telocyte.

**Fig 3:**
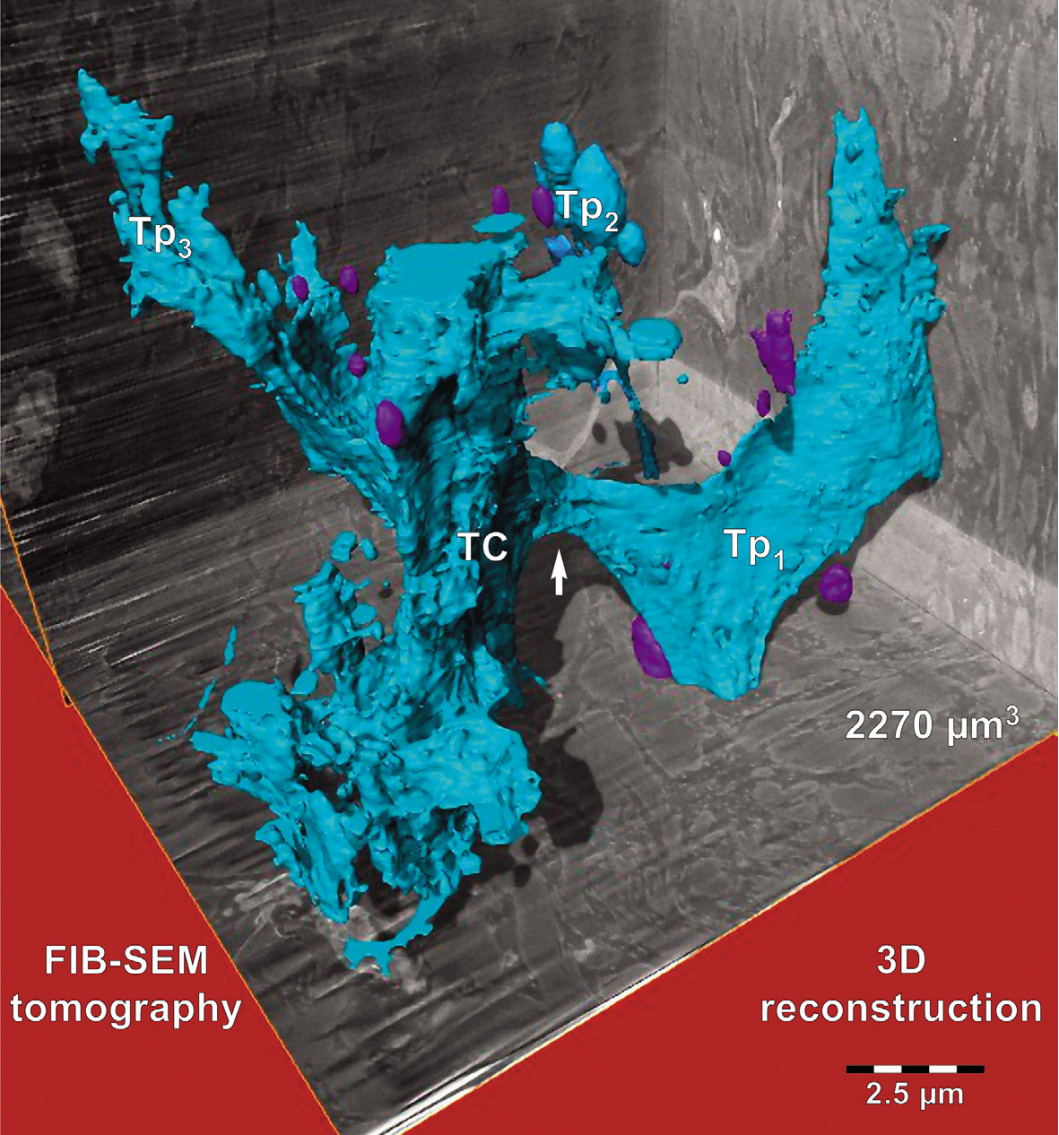
FIB-SEM tomography of a 2270 μm^3^ volume from human papillary dermis encompasses a segment from a telocyte reconstructed in blue. Three dimensional reconstruction of the stack containing the telocyte shows a ‘wing-like’ telopode (Tp1), a telopode (Tp2) with typical appearance (details in Fig.4) and a telopode (Tp3) with anfractuous contour. The arrow indicates the narrow emergence of Tp1 suggested by serial imaging in Figure2. A portion of the cell body (TC) is located in the centre. At least 10 extracellular vesicles appear reconstructed in purple.

**Fig 4:**
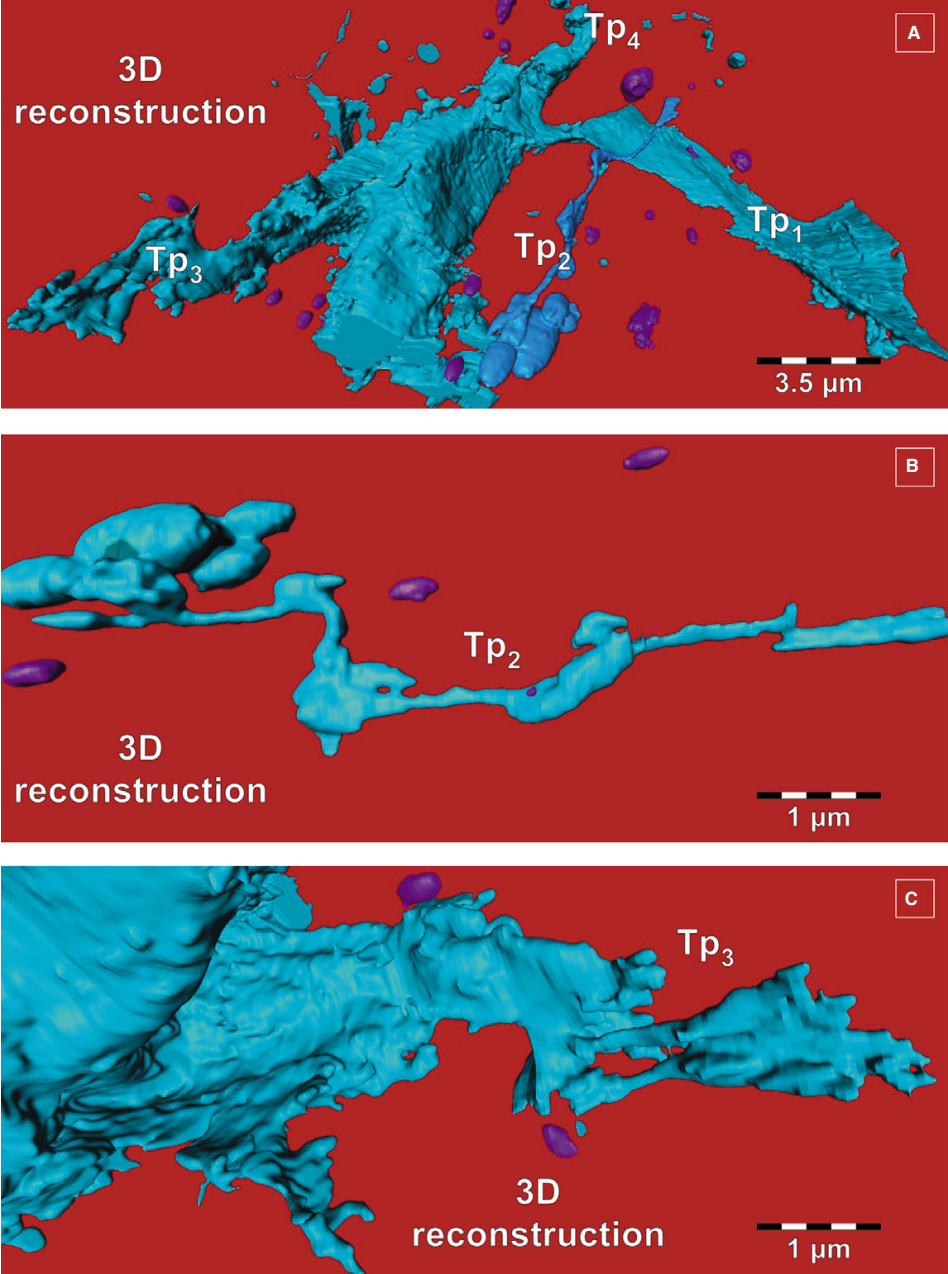
(A–C) FIB-SEM tomography. Three dimensional reconstruction details of telopodes from Figure3, from different viewing angles. (A) From this angle, a fourth telopode (Tp4) can be seen. (B) Tp2 from Figure3 has enlarged segments (podoms) alternating with slender segments. (C) Telopode (Tp3 from Fig.3) with anfractuous contour. Extracellular vesicles appear in purple.

The specific morphological features of human dermal TCs are the telopodes, as demonstrated previously using TEM 24. Figure1A depicts a TC from the papillary dermis situated just beneath the basement membrane of epidermis in close proximity with a Merkel cell. This 2D image reveals the ‘classic’ morphology of a TC: a stellate body and very thin and narrow TPs, some which appear discontinuous because of the limitations of a single plane of section. For this reason, FIB-SEM technology (ZEISS Auriga Crossbeam system) was used, which allowed imaging of several hundred serial sections and accurate reconstruction of the TC 3D volume. Automated serial sectioning and imaging of human dermis provided a total of 350 micrographs. Sixty-six images were serially removed from the block face. A stack of 275 serial images (Fig.2) were assembled to obtain a 3D reconstruction, 360° orthogonal rotation and a 3D digitally- coloured volume rendering of the TC in dermis. FIB-SEM images from Figure2 revealed the presence of a typical TC in human dermis. The same cell was clearly visible from section 67–342 allowing the reconstruction of 2270 μm^3^ (Fig.3). The TC appearance shows a cell with different extensions: a ‘ribbon-like’ telopode, a telopode with classical morphology and a telopode with anfractuous shape (details in Fig.4).

The surface-to-volume ratio is increased several folds in flat Tps compared to tubular Tps. This means, *inter alia*, a larger surface for receiving signals from extracellular space or *vice versa*. Interestingly, the dynamics of telopodes in cell culture depend on the extracellular type of matrix proteins. The stronger adherence and spreading were noted for TC seeded on fibronectin, while the lowest were on laminin 54. Moreover, in cell cultures, low-level laser stimulation (using neodymium-doped yttrium aluminium garnet laser) determines a maximum growth rate of Tp lateral extensions of 10.3 ± 1.0 μm/min. 55. This raises the possibility of using low-level laser stimulation for therapeutic purposes.

**Fig 5:**
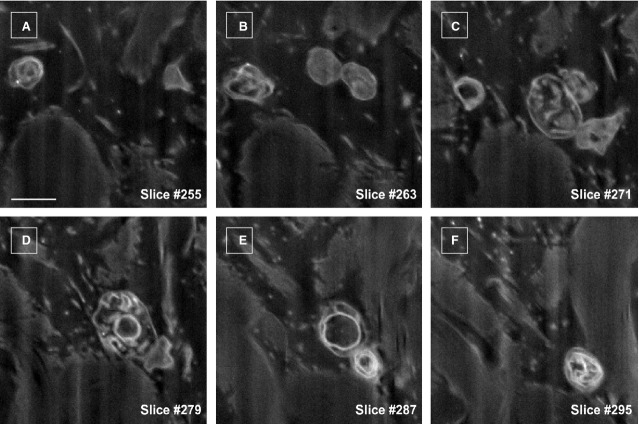
FIB-SEM of extracellular vesicles dynamics around a telocyte. Scale bar is 0.5 μm.

**Fig 6:**
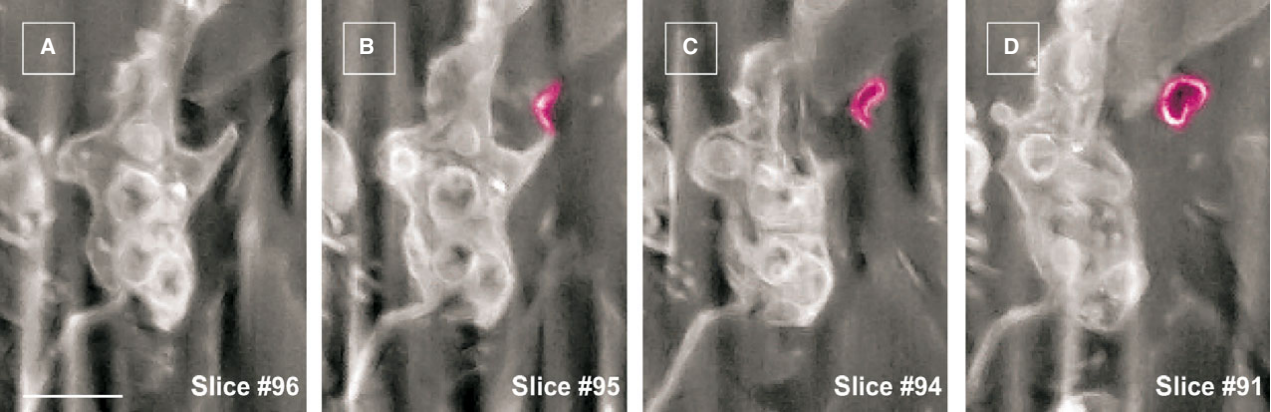
FIB-SEM of a human dermal telocyte presenting an extracellular vesicle (purple) budding from a podom. Note the empty appearance of the vesicle content. Scale bar is 0.5 μm.

The 3D reconstruction by FIB-SEM tomography of human dermal TCs allowed also the identification of extracellular vesicles (Figs.3–6, Video S1), as shown previously for cardiac TCs 56.

In fact, a rough estimation of the number of extracellular vesicles (*n* = 30 for one cell) showed a vesicle diameter of 438.6 ± 149.1 nm. Considering the international standards *e.g*. (*i*) dimensions over 100 nm, (*ii*) origin by budding and shedding of plasma membrane and (*iii*) the monovesicular ultrastructure) 57,58, we think that the extracellular vesicles we found by FIB-SEM are microvesicles or ectovesicles or shed vesicles, rather than exosomes.

As previously shown, TCs were found in human dermis having a strategic position: around blood vessels, in the perifollicular sheath, outside the glassy membrane and surrounding sebaceous glands, arrector pili muscles and both the secretory and excretory segments of eccrine sweat glands 24. Moreover, TCs frequently co-exist in close contacts with stem cells, for example, in skin dermis 24, lungs 2, skeletal muscle 59, meninges and choroid plexus 4 or liver 7. Therefore, we consider that TCs together stem cells form a structural and functional unit, a ‘tandem’ 18. This opinion is supported by the fact that TCs transfer extracellular vesicles loaded with microRNAs to stem cells 21, as well as the fact that extracellular vesicles have potential roles in regenerative medicine 60.

Last but not least, very recent data suggest that TCs through their Tps could be regarded as a primitive nervous system 61 or being involved in morphogenetic bioelectrical signalling 62,63. Telocytes are expected to contribute to age-intervention protocols 64.

## Supporting Information

**Video S1.** Movie presenting a vesicle budding from a podom.
